# An Adaptive Cultural Algorithm with Improved Quantum-behaved Particle Swarm Optimization for Sonar Image Detection

**DOI:** 10.1038/s41598-017-17945-3

**Published:** 2017-12-18

**Authors:** Xingmei Wang, Wenqian Hao, Qiming Li

**Affiliations:** 1 College of Computer Science and Technology, Harbin Engineering University, 145 Nantong Street, Harbin, Heilongjiang Province 150001 China; 2 Institute of Acoustics, Chinese Academy of Science, Beijing, 10080 China

## Abstract

This paper proposes an adaptive cultural algorithm with improved quantum-behaved particle swarm optimization (ACA-IQPSO) to detect the underwater sonar image. In the population space, to improve searching ability of particles, iterative times and the fitness value of particles are regarded as factors to adaptively adjust the contraction-expansion coefficient of the quantum-behaved particle swarm optimization algorithm (QPSO). The improved quantum-behaved particle swarm optimization algorithm (IQPSO) can make particles adjust their behaviours according to their quality. In the belief space, a new update strategy is adopted to update cultural individuals according to the idea of the update strategy in shuffled frog leaping algorithm (SFLA). Moreover, to enhance the utilization of information in the population space and belief space, accept function and influence function are redesigned in the new communication protocol. The experimental results show that ACA-IQPSO can obtain good clustering centres according to the grey distribution information of underwater sonar images, and accurately complete underwater objects detection. Compared with other algorithms, the proposed ACA-IQPSO has good effectiveness, excellent adaptability, a powerful searching ability and high convergence efficiency. Meanwhile, the experimental results of the benchmark functions can further demonstrate that the proposed ACA-IQPSO has better searching ability, convergence efficiency and stability.

## Introduction

Sonar imaging has the advantages of long-distance detection and strong penetration. Therefore, it is being extensively used for underwater inspections, hydrographic and bathymetric surveys, underwater positioning, dredging, cable-laying, pipe-line inspections, and numerous other marine applications. The underwater sonar image contains three kinds of regions, including object-highlight, shadow and background regions. The purpose of object detection is to segment the object-highlight and shadow regions from complex background region and preserve as much of the original edge information of underwater sonar image as possible^1^. A number of techniques have been proposed to detect underwater objects of the sonar image. A fuzzy clustering method on the sonar image has been proposed to solve the detection problem^2^. However, fuzzy clustering is very sensitive to speckle noise. A Markov segmentation algorithm was used for three-class sonar image detection^3^. Later, many improved image detection algorithms on Markov random field model were proposed by scholars^4^. Although their results are satisfactory, the processing procedures are quite complex and computationally costly. Maria Lianantonakis and Yvan R. Petillot developed active contours and level set methods, which were applied to the detection of regions like the seabed^5^. Subsequently, Implicit Active Contours were used in sonar image detection^6^. On this basis, Xiu-FenYe *et al*. proposed a new detection method of sonar images, Gauss-Markov random field model was integrated into level set energy function model to dynamically extract regions of interest^7^. Wang Xingmei *et al*. presented a narrowband Chan-Vese model by adaptive ladder initialization to precisely and quickly detect underwater objects of sonar images^8^. However, sometimes if the block mode k-means clustering algorithm cannot quickly and accurately complete initial segmentation, approximate position of object-highlight and shadow regions will not be obtained, and higher detection precision will not be possible.

In recent years, the cultural algorithm (CA) has gradually attracted more global attention. It can provide a powerful framework for the solution of complicated problems^9^. Youlin Lu *et al*. proposed a hybrid multi-objective cultural algorithm to solve short-term hydrothermal scheduling problems, which combined the differential evolutionary algorithm with CA framework^10^. It can obtain a more accurate solution. Later, Zhou Wei *et al*. presented the cultural particle swarm optimization algorithm (CPSO) to solve the partner selection problems of virtual enterprise^11^. The proposed algorithm has some feasibility and efficiency. To solve the optimization problems, Noor H. Awad *et al*. proposed a CA with an improved local searching algorithm^12^. Compared with the basic CA, the performance of this algorithm is greatly improved. Mostafa Z. Ali *et al*. combined the niche algorithm with the Tabu search algorithm in CA to solve engineering optimization problems, which was efficient and robust to some extent^13^. Tianyu Liu *et al*. introduced the QPSO into CA to solve multiobjective optimization problems^14^. The proposed algorithm has high efficiency. Through the comparative analysis, many intelligent optimization algorithms can be used as evolution strategy in the population space, which can increase population diversity, improve the searching ability and promote efficiency. Among intelligent optimization algorithms, QPSO is one of most commonly used algorithms in the population space of CA. This algorithm supposes that particles have quantum behaviour such that particles are attracted by a quantum potential well centered on its local attractor. It has fewer parameters and a relatively good searching ability^15^. The contraction-expansion coefficient is the only parameter in the QPSO algorithm, which plays an important role in balancing the global and local searching abilities. However, it is a random value, which easily leads to blindness in the searching process. To solve this problem, the revised QPSO algorithm regarded iterative times as an important factor to adjust the contraction-expansion coefficient^16^. The contraction-expansion coefficient can linearly decrease with the increase of iterative times in the interval [0.5 1). Although this method is often used in practice, it only solves the linear problem and easily falls into the local optimal solution in the searching process of complex problems. Subsequently, in order to improve the performance of the QPSO algorithm, Jun Sun *et al*. further presented a diversity-maintained QPSO algorithm^17^. When the population diversity is lower than a set value, contraction-expansion coefficient is set as the boundary of convergence, or contraction-expansion coefficient linearly decreases. On this basis, Tian Jin constructed a new contraction-expansion coefficient using Sigmoid function to solve high-dimensional multimodal functions optimization problems in the QPSO algorithm^18^. This method can make the contraction-expansion coefficient decrease nonlinearly with the iterative times and increase the flexibility of the QPSO algorithm. Although these adjustments of the contraction-expansion coefficient in the QPSO algorithm can to some extent improve searching ability, these algorithms only regard iterative times as the factor to adjust the contraction-expansion coefficient. In fact, the quality of particles also influences their searching ability. Therefore, the fitness value is an important factor to adjust the contraction-expansion coefficient.

In addition, the communication protocol between population space and belief space can influence the performance of CA. Accept function is used to set the rate of accepted individuals in the population space, and the rate of accepted individuals usually decreases with iterative times. Ricardo Landa Becerra and Carlos A.Coello improved accept function by resetting the rate of accepted individuals when the best solution had not changed over the last several iterations^19^. Since this method can improve the performance of CA, it is widely used in CAs^20,21^. However, appropriate range of rate is different when solving different problems, the rate of accepted individuals still need to be reset. To enhance the flexibility, the fitness values of individuals were used in accept function^22^. Although this adjustment can somewhat enhance the flexibility, the utilization of evolution information was not adequately used. Influence function is used to guide the evolution of poor individuals in the population space by the knowledge in the belief space. Different types of knowledge are chosen according to different problems^23,24^. These types of knowledge can determine the searching step size and searching direction of the individuals in the population space.

In these regards, to obtain more accurate detection results, this paper presents an ACA-IQPSO to detect underwater sonar image. In the population space, the contraction-expansion coefficient of the IQPSO is adaptively adjusted according to iterative times and the fitness value of particles. In the belief space, a new update strategy is adopted to update the cultural individuals using the idea of SFLA. In addition, accept function and influence function are redesigned in the new communication protocol. The new communication protocol can make belief space with adequate evolutionary information that can more precisely guide the evolution of particles in the population space and further improve the searching ability of the algorithm. The new communication protocol can enhance convergence efficiency of the algorithm. The experimental results demonstrate that the ACA-IQPSO can locate good clustering centres according to the grey distribution information of underwater sonar images, and accurately complete underwater object detection. Through the analysis of benchmark functions, it can show that the proposed ACA-IQPSO is significantly better than other algorithms in searching ability, convergence efficiency and stability. Therefore, the proposed method has important theoretical and practical value.

## Methods

### CA and QPSO

#### CA

**Figure 1:**
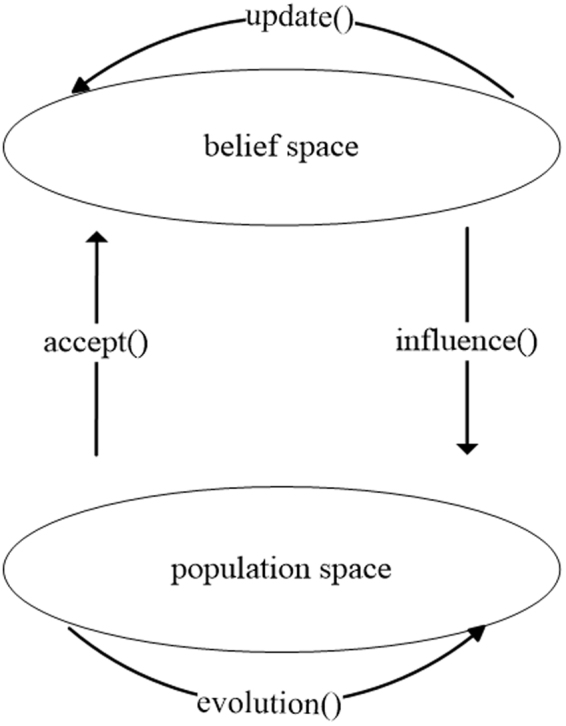
Schematic diagram of CA.

CA is a model with double level evolutionary space. It defines both population space and belief space. The two spaces evolve respectively, and the communication protocol between these spaces is accomplished by accept function and influence function. The schematic diagram is shown in Fig. 1. In the population space, the intelligent optimization algorithm can be used as evolution strategy to achieve the evolution of individuals. These individuals in the population space can contribute their experience and evolution information to the belief space through accept function. In the belief space, the experience and evolutionary information are converted into the cultural individuals. And the knowledge is extracted from the cultural individuals to guide the evolution of individuals in the population space. There are five types of knowledge in the belief space, including situational knowledge, normative knowledge, historical knowledge, topographical knowledge and domain knowledge. These types of knowledge are used to influence the evolution of poor individuals in the population space by influence function, and guide individuals to generate elite offspring.

#### QPSO

The QPSO supposes that particles have quantum behaviour, and they move in a quantum potential well centred on its local attractor. When one particle finds a local optimal state, some particles quickly converge to it, and others appear on any position of the whole space in a certain probability. Particles with quantum-behaved maintain high levels of population diversity, which can avoid falling into the local optimal solution to a certain extent. In addition, it only updates the position without speed. Therefore, the QPSO has relatively fewer parameters and a good searching ability.

In the D-dimensional space, *N* is the population size. The particle swarm is defined as *X* = [*X*_1_, *X*_2_, *X*_3_, ... *X*_*N*_], and *X*_*i*_ = [*X*_*i*1_, *X*_*i*2_, *X*_*i*3_, ... *X*_*iD*_] represents the current position of the *i* th particle. The position update of particles is shown as:

1 $${X}_{i}(t+1)=\{\begin{array}{ll}{P}_{m}(t)+\frac{L(t)}{2}\,\mathrm{ln}(\frac{1}{\mu }) & \mu  < 0{\rm{.5}}\\ {P}_{m}(t)-\frac{L(t)}{2}\,\mathrm{ln}(\frac{1}{\mu }) & otherwise\end{array}$$

where *μ* is a random number in the interval [0,1], *P*_*m*_(*t*) is the local attractor, *L*(*t*) is the characteristic length of the wave function, and *t* is the current iterative times.

*P*_*m*_(*t*) is defined as:

2 $${P}_{m}(t)=\phi \cdot {P}_{i}(t)+(1-\phi )\cdot G(t)\quad \quad \phi \in (0,1)$$

where *P*_*i*_(*t*) is the personal best position of the particle, and *G*(*t*) is the global best position.

*L*(*t*) is expressed as:

3 $$L(t)=2\beta \cdot |mbest(t)-{X}_{i}(t)|$$

where *β* is the contraction-expansion coefficient, which can control the convergence speed of the algorithm. The contraction-expansion coefficient *β* has an important influence on the searching ability of particles. *mbest* (*t*) is the mean best position of all particles.

*mbest*(*t*) is given by:

4 $$\begin{array}{rcl}mbest(t) & = & [{m}_{1}(t),{m}_{2}(t),\mathrm{...}{m}_{D}(t)]\\  & = & [\frac{1}{N}\sum _{i=1}^{N}{P}_{i1}(t),\frac{1}{N}\sum _{i=1}^{N}{P}_{i2}(t),\mathrm{...}\frac{1}{N}\sum _{i=1}^{N}{P}_{iD}(t)]\end{array}$$

According to Eq. (1) and Eq. (3), the position of the particles can be expressed as:

5 $${X}_{i}(t+1)=\{\begin{array}{ll}{P}_{m}(t)+\beta \cdot |mbest(t)-{X}_{i}(t)|\cdot \,\mathrm{ln}(\frac{1}{\mu }) & \mu  < 0.5\\ {P}_{m}(t)-\beta \cdot |mbest(t)-{X}_{i}(t)|\cdot \,\mathrm{ln}(\frac{1}{\mu }) & otherwise\end{array}$$

### The proposed ACA-IQPSO

#### Population space

The IQPSO is integrated into CA as the evolution strategy of the population space in the ACA-IQPSO.

IQPSO: In the QPSO, the quality of particles influences their behaviour in the searching process. The contraction-expansion coefficient is an important factor to control the particles’ behaviour. It only regards iterative times as the factor to generally adjust the contraction-expansion coefficient. However, this method easily falls into the local optimal solution in the searching process of complex problems. Furthermore, the quality of particles depends on information carried by themselves in each iteration, and all the information also has important influence on particles’ behaviour in the searching process. Therefore, iterative times and the fitness value of particles are used to adaptively adjust the contraction-expansion coefficient in IQPSO. In each iteration, when the quality of particles is worse, the contraction-expansion coefficient is larger, and the global searching ability of the particles is relatively stronger. When the quality of particles is better, the contraction-expansion coefficient is relatively smaller, and the local searching ability of the particles is stronger.

The contraction-expansion coefficient *β* is defined as follows:

6 $$\beta =\frac{1}{1+\frac{T-t}{T\cdot k}\cdot {e}^{\frac{-{|{f}_{g}-{f}_{i}|}^{2}}{2\cdot {(\bar{f}-{f}_{w})}^{2}}}}$$

where *T* is the maximum iterative times, *f*_*g*_ is the fitness value of *G*(*t*), $\bar{f}$ is the average fitness value of particles in the population space, *f*_*i*_ is the fitness value of the *i* th particle, and *f*_*w*_ is the fitness value of the worst particle. *k* is a positive integer, that can adjust *β* to balance the relationship between iterative times and the fitness value of particles.

**Figure 2:**
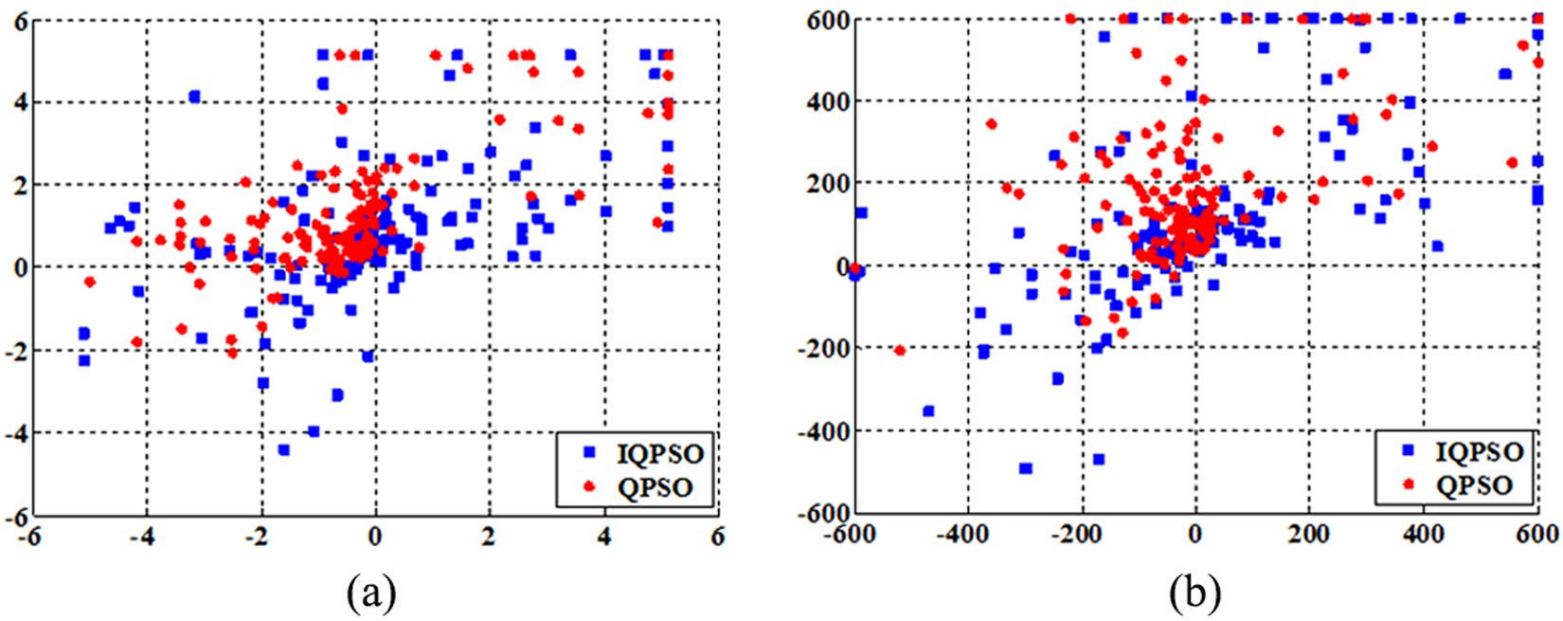
Position distribution of particles in IQPSO and QPSO. (**a**) Position distribution of particles on Sphere function. (**b**) Position distribution of particles on Griewank function.

The performance analysis of IQPSO: To verify the superiority of the IQPSO in searching ability, Sphere function and Griewank function are used in this paper to test position distribution of particles. Sphere function is unimodal and contains only one global optimal solution. Griewank function is multimodal and contains many local optimal solutions, but only one global optimal solution. Fig. 2 shows position distribution of particles in IQPSO and QPSO^16^. The relevant parameters are as follows. The dimension of the solution space is 2, the population size is 30, and the maximum number of iterations is 5.

As seen from Fig. 2, the solution scope is relatively larger in the IQPSO, and IQPSO more easily obtains the global optimal solution. Therefore, compared with the QPSO, the IQPSO can increase population diversity and improve searching ability.

**Figure 3:**
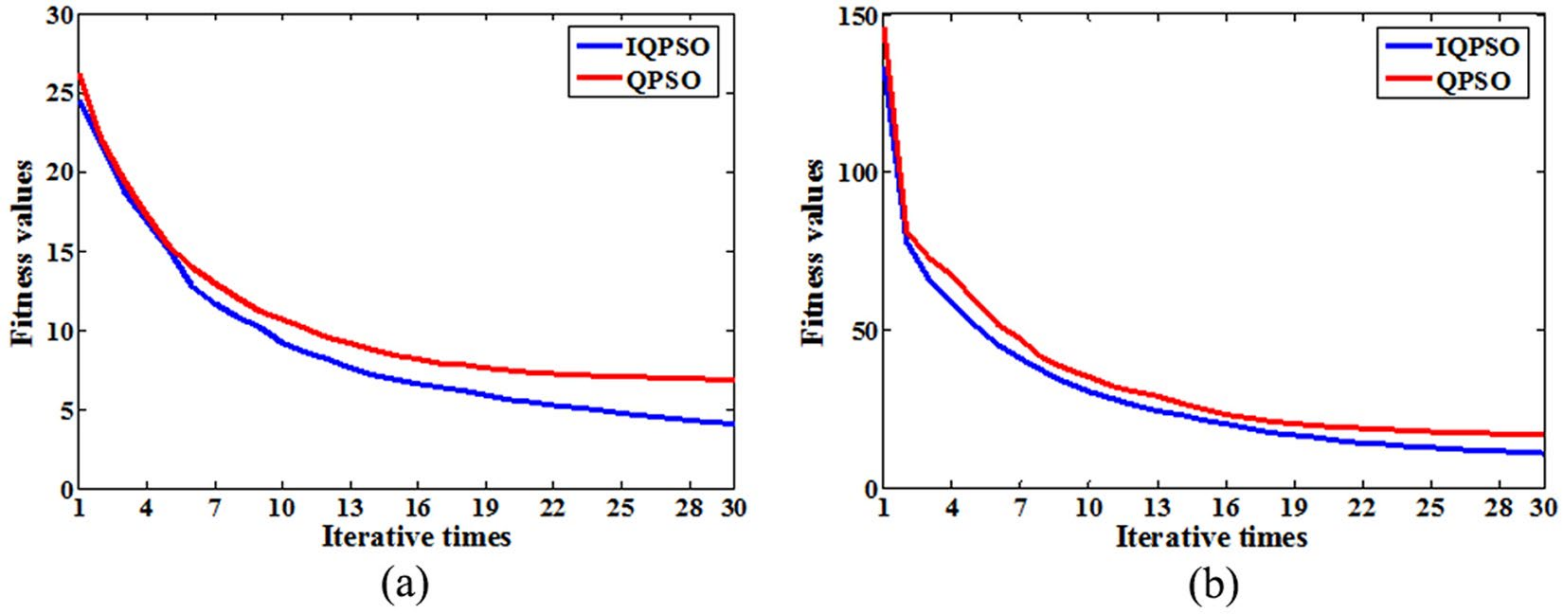
The optimization results in IQPSO and QPSO. (**a**) The optimization results of Sphere function. (**b**) The optimization results of Griewank function.

To further verify the effectiveness of the IQPSO in searching ability, the fitness values are calculated by Sphere and Griewank functions in the IQPSO and the QPSO. The optimization results are shown in Fig. 3. The relevant parameters are as follows. The dimension of the solution space is 10, the population size is 30, the maximum number of iterations is 30, and the experiment runs for 30 times in each algorithm.

As depicted in Fig. 3, whether it is unimodal function or multimodal function, the fitness values of the IQPSO are always smaller than the QPSO in the iterative process. Therefore, the optimal solutions of the IQPSO are always better than the QPSO. The optimization results further show that the searching ability of the IQPSO is better than the QPSO.

#### Belief space

In the ACA-IQPSO, the cultural individuals in the belief space are updated by a new update strategy. Meanwhile, three types of knowledge are extracted from the cultural individuals. These types of knowledge are used to influence the evolution of particles in the population space by influence function, and guide particles to generate elite offspring.

Update strategy: Cultural individuals are updated by selection, crossover and mutation in many CAs^11^. However, this update strategy needs many parameters that make the algorithm not easy to control and lead to poor searching ability. In SFLA, the worst frog is updated by the step size that is the difference between the best frog and the worst frog. This update strategy has few parameters, powerful searching ability and is easy to implement^25^. Therefore, a new update strategy is adopted to update the cultural individuals in the belief space according to the idea of the update strategy in SFLA. The new update strategy adopts the difference between the situational knowledge and the cultural individuals as step size. New update strategy is expressed as follows:

7 $${Y}_{j}(t+1)={Y}_{j}(t)+rand\cdot (S(t)-{Y}_{j}(t))$$

where *Y*_*j*_(*t*) is the position of the *j* th cultural individual, *S*(*t*) is situational knowledge of the *t* th iteration in belief space, and *rand* is a random number in the interval [0,1].

Knowledge structure: Situational knowledge, normative knowledge and domain knowledge are adopted in the belief space of ACA-IQPSO. In each iteration, these types of knowledge are updated by cultural individuals.

Situational knowledge contains the best cultural individual in the belief space. In each iteration, when the fitness value of the best cultural individual is larger than that of the current situational knowledge, the best cultural individual is used as situational knowledge in the update process. Update formula of situational knowledge is:

8 $$S(t+1)=\{\begin{array}{ll}S(t) & {f}_{S}(t) < {f}_{yg}(t)\\ {Y}_{g}(t) & otherwise\end{array}$$

where *Y*_*g*_(*t*) is the best cultural individual in the *t* th iteration, *f*_*S*_(*t*) is the fitness value of *S*(*t*), and *f*_*yg*_(*t*) is the fitness value of *Y*_*g*_(*t*).

Normative knowledge describes the feasible solution space of the problem in the belief space. It stores the boundary information that can guide particles in the population space to search for a better region. The update of normative knowledge reflects the change of the feasible search space. With the increase of iterative times, the searching scope can be concentrated in the dominant region. Therefore, when an excellent cultural individual is out of the current searching scope in each iteration, normative knowledge is updated. Update formula of normative knowledge is as follows:

9 $${f}_{l}(t+1)=\{\begin{array}{ll}{f}_{j}(t) & {f}_{j}(t) < {f}_{l}(t)\\ {f}_{l}(t) & otherwise\end{array}$$

10 $${f}_{u}(t+1)=\{\begin{array}{ll}{f}_{j}(t) & {f}_{j}(t) > {f}_{u}(t)\\ {f}_{u}(t) & otherwise\end{array}$$

where *Y*_*j*_(*t*) is the position of the *j* th cultural individual, *f*_*j*_(*t*) is the fitness value of *Y*_*j*_(*t*), *f*_*l*_(*t*) is lower limit of fitness value in the *t* th iteration, *f*_*u*_(*t*) is upper limit of fitness value in the *t* th iteration.

Domain knowledge is used to statically or dynamically guide the particles in the population space to evolve along the predictive direction, and record a good evolutionary trend. In the belief space, the centre of gravity can reflect the overall distribution of the cultural individuals and guide the particles in the population space to search better solutions. The evolutionary direction of the particles in the population space can be predicted by updating the centre of gravity in each iteration, which can improve the searching efficiency. Therefore, domain knowledge stores the centre of gravity in the belief space to guide the evolution of particles in the population space in this paper. The centre of gravity *GT*(*t*) is defined as follows:

11 $$\begin{array}{rcl}GT(t) & = & [G{T}_{1}(t),G{T}_{2}(t),\mathrm{...}G{T}_{D}(t)]\\  & = & [\frac{1}{M}\sum _{j=1}^{M}{Y}_{j1}(t),\frac{1}{M}\sum _{j=1}^{M}{Y}_{j2}(t),\mathrm{...}\frac{1}{M}\sum _{j=1}^{M}{Y}_{jD}(t)]\end{array}$$

where *M* is the size of cultural individuals, and *D* is the dimension of cultural individuals.

#### New communication protocol

The communication protocol between the population and belief spaces is accomplished by accept function and influence function. To enhance utilization of information in the population and belief spaces, accept and influence functions are redesigned in the new communication protocol.

Accept function: Accept function is used to set the rate of accepted particles in the population space. To further improve the flexibility of the rate, accept function is redesigned in this paper. The new accept function can adaptively change the rate of accepted particles according to the quality of particles in each iteration. Meanwhile, it can enhance utilization degree of information in the population space. When the quality of particles is better, the accept rate is larger. The new accept function is defined as follows:

12 $$Af=\frac{\bar{f}-{f}_{w}}{\sum _{i=1}^{N}|{f}_{i}-\bar{f}|}$$

where $\bar{f}$ is the average fitness value of particles in the population space, *f*_*w*_ is the fitness value of the worst particle in the population space, and *f*_*i*_ is the fitness value of the *i* th particle.

Therefore, the number of accepted particles in population space is calculated according to Eq. (12). It is:

13 $$num=\lfloor N\cdot Af\rfloor $$

where *N* is the population size.

For new accept function, Eq. (12) is analysed in detail. The fitness values of particles in population space are sorted in descending order. Therefore, if there is a position *c* where the fitness value *f*_*c*_ is larger than $\bar{f}$, and the fitness value *f*_*c* + 1_ is smaller than $\bar{f}$. *Af* is:

14 $$\begin{array}{rcl}Af & = & \frac{\bar{f}-{f}_{w}}{\sum _{i=1}^{N}|\,{f}_{i}-\bar{f}|}\\  & = & \frac{\bar{f}-{f}_{N}}{({f}_{1}+{f}_{2}+\mathrm{...}+{f}_{c}-c\cdot \bar{f})+(N-c)\cdot \bar{f}-({f}_{c+1}+\mathrm{...}+{f}_{N})}\\  & = & \frac{\bar{f}-{f}_{N}}{N\cdot \bar{f}-2\cdot A+(N-2\cdot c)\cdot \bar{f}}\\  & = & \frac{1}{2}\cdot \frac{\bar{f}-{f}_{N}}{(N-c)\cdot \bar{f}-A}\end{array}$$

where *c* is an integer in the interval [1, *N* − 1], *A* = *f*_*c* + 1_ + ... + *f*_*N*_, and *A* ≥ (*N* − *c*) . *f*_*N*_.

Therefore, Eq. (15) is as follows:

15 $$Af=\frac{\bar{f}-{f}_{w}}{\sum _{i=1}^{N}|{f}_{i}-\bar{f}|}\le \frac{1}{2}\cdot \frac{1}{N-c}$$

As seen from Eq. (15), when *c* = *N* − 1, the maximum value of *Af* is 0.5. When *c* is relatively larger, there are more particles whose fitness values are larger than the average fitness value, and the quality of particles in the population space is better. Correspondingly, more particles are accepted. When *c* is relatively smaller, there are fewer particles whose fitness values are larger than the average fitness value, and the quality of particles in the population space is worse. Correspondingly, fewer particles are accepted. Therefore, new accept function is effective in this paper.

Influence function: Influence function is used to guide the evolution of poor particles in the population space by using the knowledge in the belief space. These types of knowledge determine searching step size and searching direction of the particles in the population space. To enhance utilization of information in the belief space and more precisely guide the evolution of poor particles in the population space, influence function is also redesigned in this paper. The new influence function adopts situational, normative and domain knowledge to guide the evolution of poor particles in the population space. Situational and domain knowledge are used to determine searching step size, and normative knowledge is used to determine searching direction. When the fitness values of the poor particles are less than the lower limit of fitness value in the belief space, domain knowledge is used to control searching step size. When the fitness values of poor particles are less than the upper limit of fitness value in the belief space, situational knowledge is used to control searching step size. Otherwise, the positions of poor particles are randomly generated in the solution space.

The influence function is defined as follows:

16 $${X}_{i}=\{\begin{array}{ll}(\alpha {X}_{i}+\omega (GT-{X}_{i}))/(\alpha +\omega ) & {f}_{i} < {f}_{l}\\ (\alpha {X}_{i}+\omega (S-{X}_{i}))/(\alpha +\omega ) & {f}_{l}\le {f}_{i}\le {f}_{u}\\ rand\cdot (bound1,bound2) & otherwise\end{array}$$

where *bound*1 and *bound*2 are the lower bound and upper bound of solution space respectively. *α*, *ω*, and *rand* are random number in the interval [0,1].

**Figure 4:**
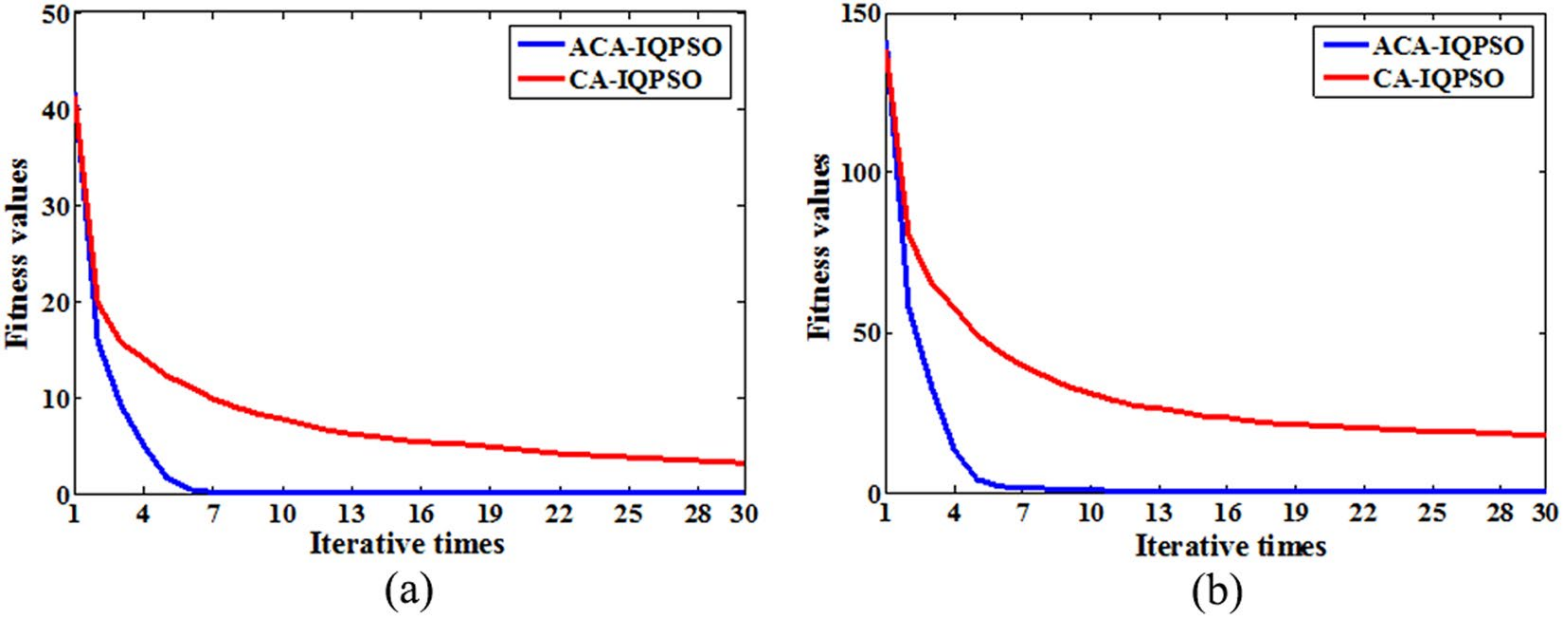
The optimization results in ACA-IQPSO and CA-IQPSO. (**a**) The optimization results of Sphere function. (**b**) The optimization results of Griewank function.

The performance analysis of new communication protocol: To verify the effectiveness of the new accept function and influence function in the ACA-IQPSO, Sphere and Griewank functions are used to compare the ACA-IQPSO with a cultural algorithm with improved quantum-behaved particle swarm optimization (CA-IQPSO). The CA-IQPSO introduces the IQPSO into CA, which uses the communication protocol in cultural algorithm for power system stabilizer (CA-PSS)^20^. The fitness values are calculated by Sphere and Griewank functions in the ACA-IQPSO and the CA-IQPSO. The optimization results are shown in Fig. 4.The relevant parameters are as follows. The dimension of the solution space is 10, the population size is 30, the maximum number of iterations is 30, the size of cultural individuals is 8, and the experiment runs for 30 times in each algorithm.

As depicted in Fig. 4, whether it is unimodal function or multimodal function, the ACA-IQPSO can converge to global optimal solution after 10 iterations, convergence speed of ACA-IQPSO is faster in the iterative process. It can indicate that convergence efficiency of the ACA-IQPSO is superior to the CA-IQPSO. Meanwhile, the fitness values of the ACA-IQPSO are smaller than the CA-IQPSO. The new communication protocol can make belief space with adequate evolutionary information that can more precisely guide the evolution of particles in the population space. Therefore, the new communication protocol can further improve searching ability remarkably.

## Results and Discussion

### Experimental results and discussion of sonar image detection

In this section, the original sonar image has serious noise. To remove some noise points and make the image smoother, Butterworth lower-pass filter is used for the noise smoothing^26^. On this basis, numerical examples are shown to validate the effectiveness and adaptability of the proposed ACA-IQPSO for sonar image detection. Meanwhile, the ACA-IQPSO is compared with CA-IQPSO, CPSO^11^, IQPSO, QPSO^16^, and PSO^27^. In addition, the fitness function is mainly used to evaluate the quality of particles in the process of sonar image detection. Therefore, a fitness function combining intra-class difference with inter-class difference is adopted in these algorithms^28^.When the fitness value is larger, the detection result is better. The relevant parameters are as follows. The number of clustering centers is 4, the population size is 20, the maximum number of iterations is 20, the acceleration coefficient is c1 = c2 = 2, the inertia weight is w = 0.8 in CPSO and PSO, size of cultural individuals is M = 8 in the ACA-IQPSO, CA-IQPSO and CPSO, and the contraction-expansion coefficient *β* linearly decreases from 1.0 to 0.5 in the QPSO.

**Figure 5:**
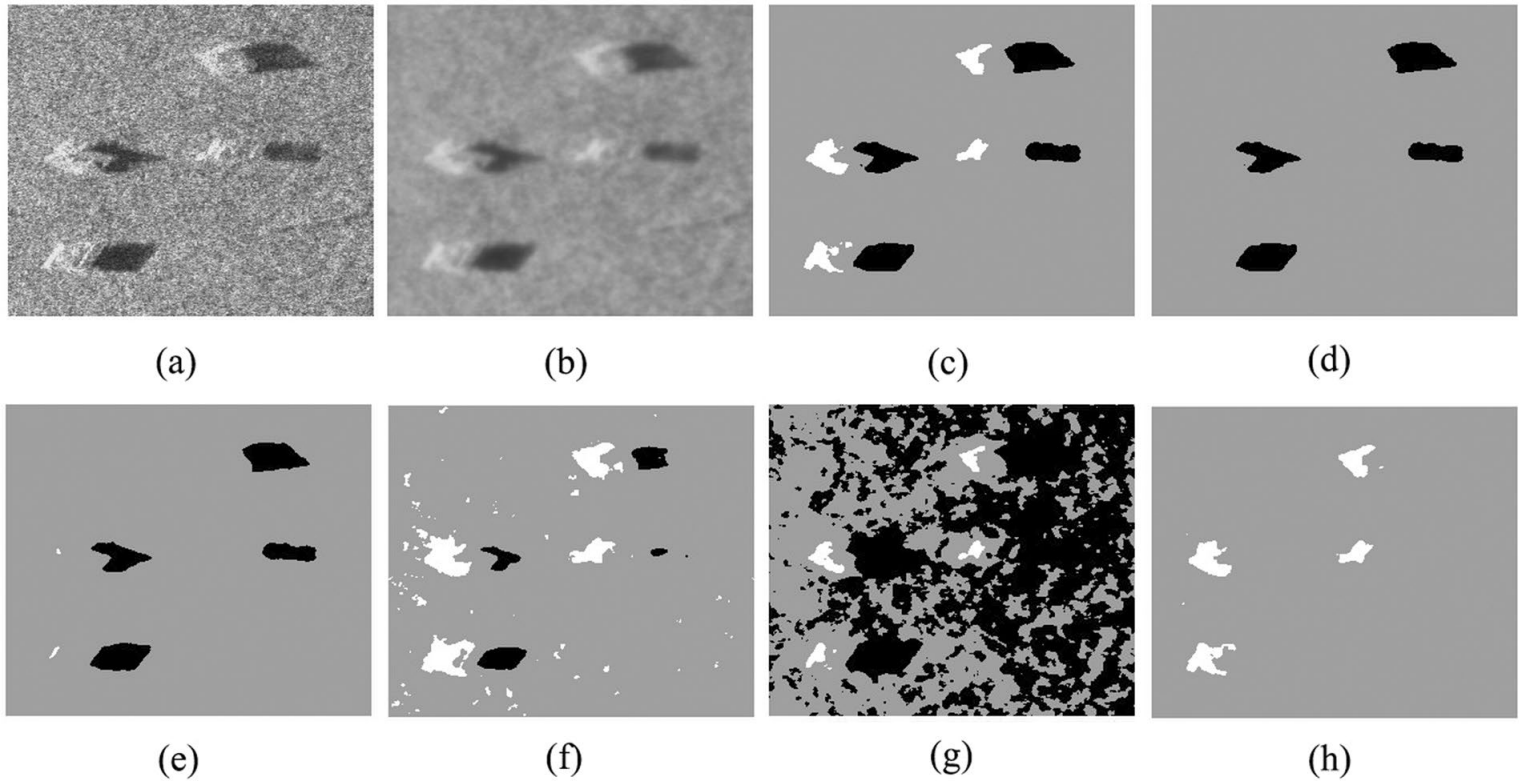
Detection results of original sonar image (image size: 277 × 325). (**a**) Original sonar image. (**b**) Smoothed image. (**c**) ACA-IQPSO. (**d**) CA-IQPSO. (**e**) CPSO. (**f**) IQPSO. (**g**) QPSO. (**h**) PSO.

Fig. 5 shows the detection results of the original sonar image with floating objects.

As depicted in Fig. 5, the proposed ACA-IQPSO can better detect object-highlight and shadow regions from complex background region. The edge information is preserved as much as possible in Fig. 5(c). While the detection result of the CA-IQPSO can only detect the shadow region in Fig. 5(d), it is not suitable for detecting the sonar image with floating objects. Although the CPSO can detect the object-highlight and shadow regions, it has serious information loss in the object-highlight region in Fig. 5(e). At the same time, IQPSO can also detect the object-highlight and shadow regions, but the detection result contains a lot of noise and the integrity of the underwater object is seriously weak in Fig. 5(f). The detection result of the QPSO has serious noise, which is not an ideal detection result in Fig. 5(g). PSO can only detect the object-highlight region in Fig. 5(h).Therefore, compared with the detection results of CA-IQPSO, CPSO, IQPSO, QPSO and PSO, the proposed ACA-IQPSO can locate good clustering centres according to the grey distribution information of the underwater sonar image with floating objects and accurately complete underwater object detection.

**Figure 6:**
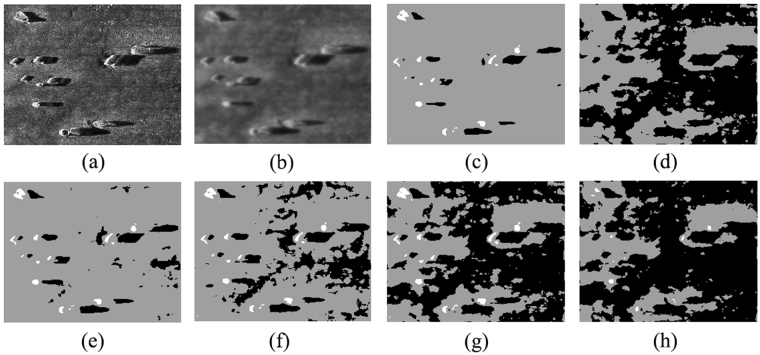
Detection results of original sonar image (image size: 203 × 257). (**a**) Original sonar image. (**b**) Smoothed image. (**c**) ACA-IQPSO. (**d**) CA-IQPSO. (**e**) CPSO. (**f**) IQPSO. (**g**) QPSO. (**h**) PSO.

**Figure 7:**
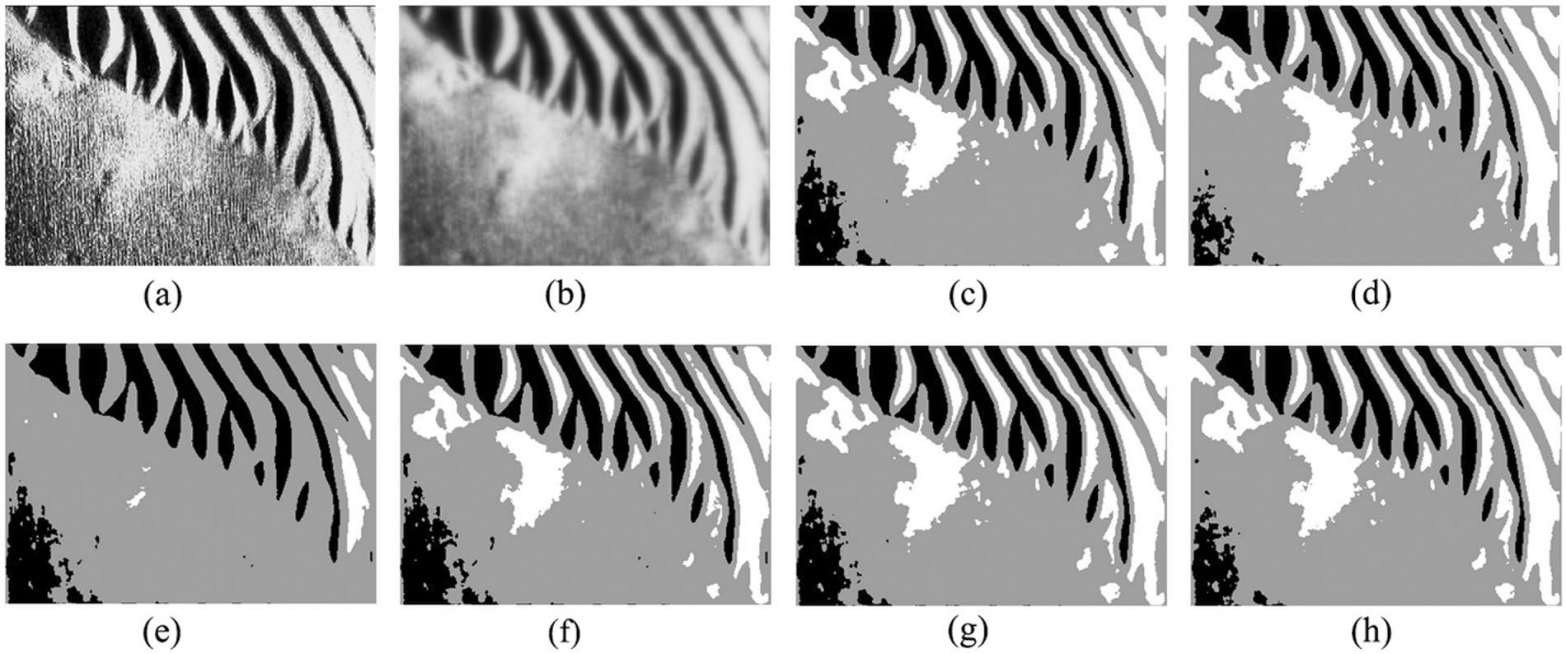
Detection results of original sonar image (image size: 259 × 368). (**a**) Original sonar image. (**b**) Smoothed image. (**c**) ACA-IQPSO. (**d**) CA-IQPSO. (**e**) CPSO. (**f**) IQPSO. (**g**) QPSO. (**h**) PSO.

**Figure 8:**
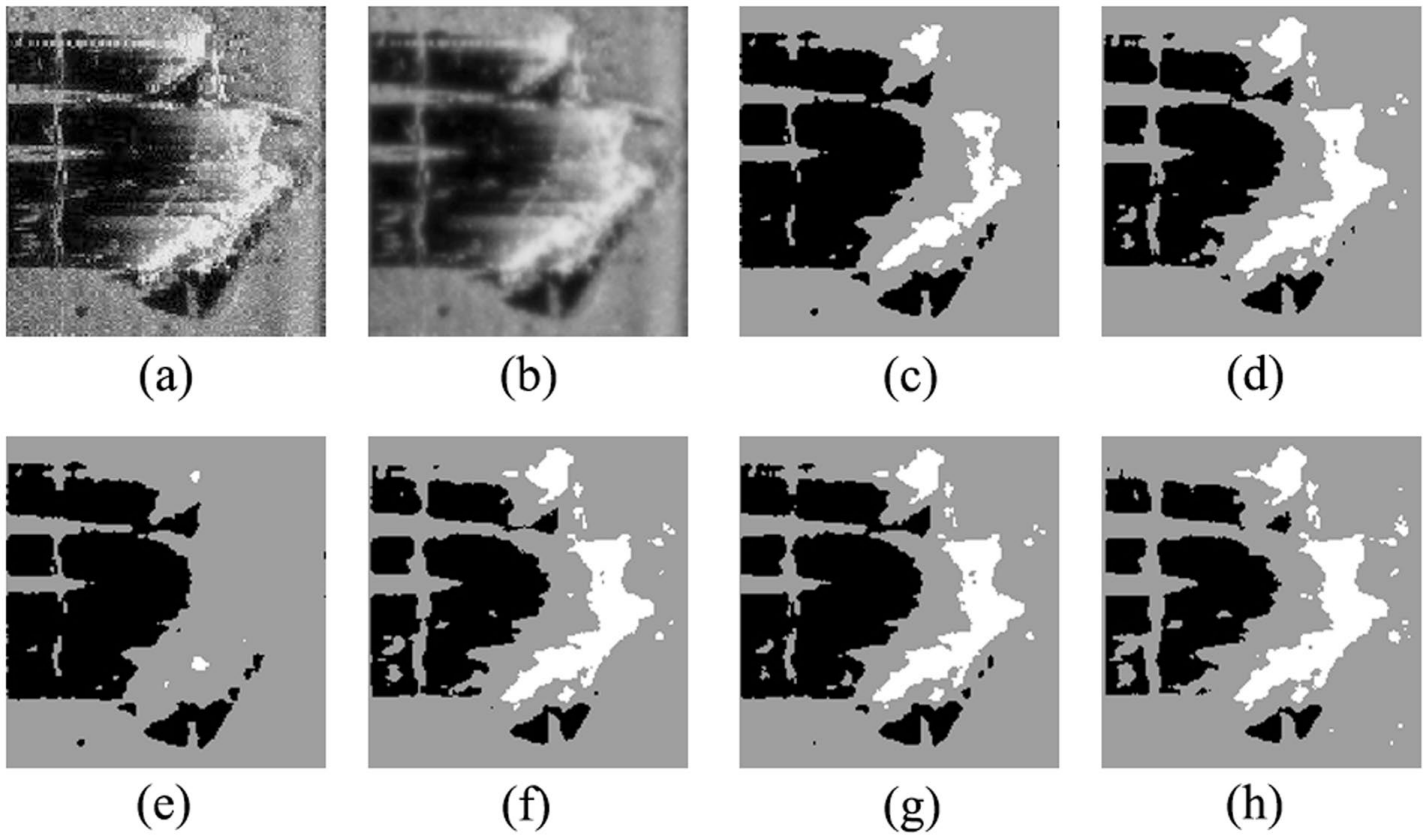
Detection results of original sonar image (image size: 173 × 167). (**a**) Original sonar image. (**b**) Smoothed image. (**c**) ACA-IQPSO. (**d**) CA-IQPSO. (**e**) CPSO. (**f**) IQPSO. (**g**) QPSO. (**h**) PSO.

To further verify the effectiveness of the proposed ACA-IQPSO, Fig. 6 shows the detection results of the original sonar image with underwater stones on the bottom, which has a relatively weak contrast. Fig. 7 shows the detection results of structured seabed that is an object in sand ripples. Fig. 8 shows the detection results of larboard original sonar image including rocks, which are partly buried in the sand.

As seen from Figs 6, 7 and 8, the proposed ACA-IQPSO can obtain relatively better detection results in Figs 6(c), 7(c) and 8(c). While Fig. 6(d) has information loss in the object-highlight region. Although Fig. 6(e),(f),(g) and (h) can detect the object-highlight region and shadow region, the detection results have serious noise, especially in Fig. 6(g) and (h). Therefore, QPSO and PSO are failed to detect the sonar image with relatively weak contrast. Meanwhile, some information is lost in the object-highlight region and shadow region in Fig. 7(d),(e),(f),(g) and (h), which is not conducive to the subsequent feature extraction and underwater object recognition. In Fig. 8, the detection results of Fig. 8(d),(f),(g) and (h) show information loss in the shadow region and over detection in object-highlight region. At the same time, the detection results have different degrees of noise. Fig. 8(e) has serious information loss in object-highlight region. They are not ideal detection results.

Through the above comparative experiments, the proposed ACA-IQPSO can obtain relatively accurate results in sonar image detection. Moreover, the detection results of the CA-IQPSO are not better than ACA-IQPSO, which further verifies the effectiveness of the new communication protocol in this paper. Meanwhile, IQPSO can relatively obtain better detection results than QPSO, this indicates that IQPSO can improve searching ability of particles.

**Figure 9:**
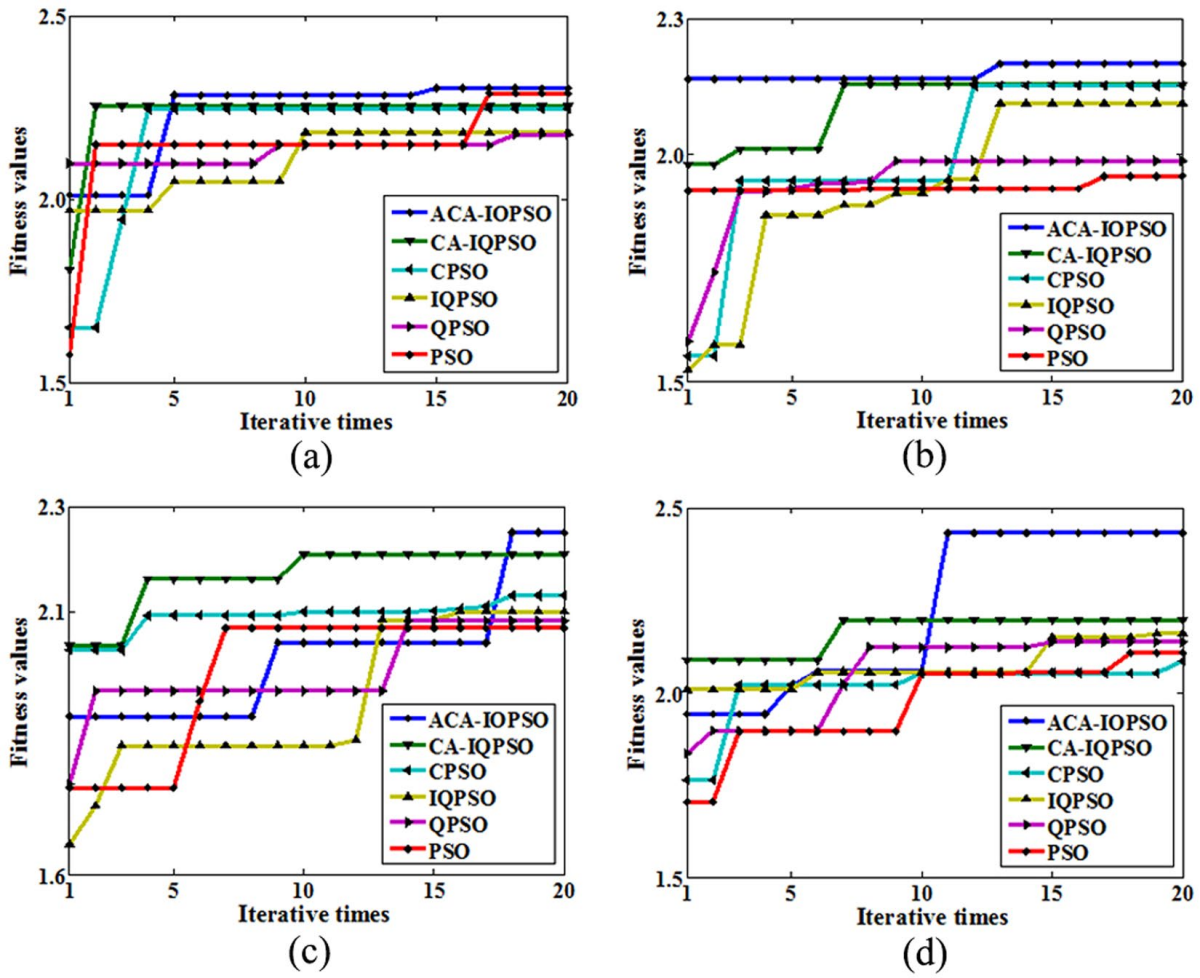
Variation of the fitness values in each iteration. (**a**) Variation of fitness values of Fig. 5. (**b**) Variation of fitness values of Fig. 6. (**c**) Variation of fitness values of Fig. 7. (**d**) Variation of fitness values of Fig. 8.

To demonstrate the advantages of proposed ACA-IQPSO more clearly, Table 1 shows the best fitness values after 20 iterations for the ACA-IQPSO and other intelligent optimization algorithms. Fig. 9 shows the variation of the best fitness values in each iteration.

**Table 1:** The best fitness values of detection results.

Image	ACA-IQPSO	CA-IQPSO	CPSO	IQPSO	QPSO	PSO
Fig. 5	2.3020	2.2544	2.2464	2.1809	2.1765	2.2887
Fig. 6	2.2001	2.1540	2.1533	2.1132	1.9859	1.9523
Fig. 7	2.2512	2.2096	2.1312	2.0992	2.0823	2.0685
Fig. 8	2.4308	2.1970	2.0857	2.1590	2.1374	2.1065

It can be seen from Table 1 and Fig. 9 that the best fitness values of the proposed ACA-IQPSO are larger than other intelligent optimization algorithms after 20 iterations, which shows the powerful searching ability and high convergence efficiency of ACA-IQPSO. Meanwhile, the best fitness values of CA-IQPSO are larger than IQPSO and the best fitness values of CPSO are larger than PSO, which can demonstrate the effectiveness of CA. Among IQPSO, QPSO and PSO, the best fitness values of IQPSO and QPSO are close, but the IQPSO is superior to the QPSO, and they are larger than PSO. These results indicate that IQPSO has the merit of searching ability.

**Figure 10:**
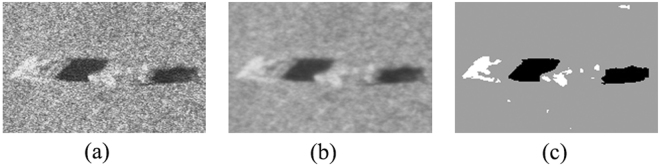
Detection results of original sonar image (image size: 130 × 201). (**a**) Original sonar image. (**b**) Smoothed image. (**c**) Detection result of ACA-IQPSO.

**Figure 11:**
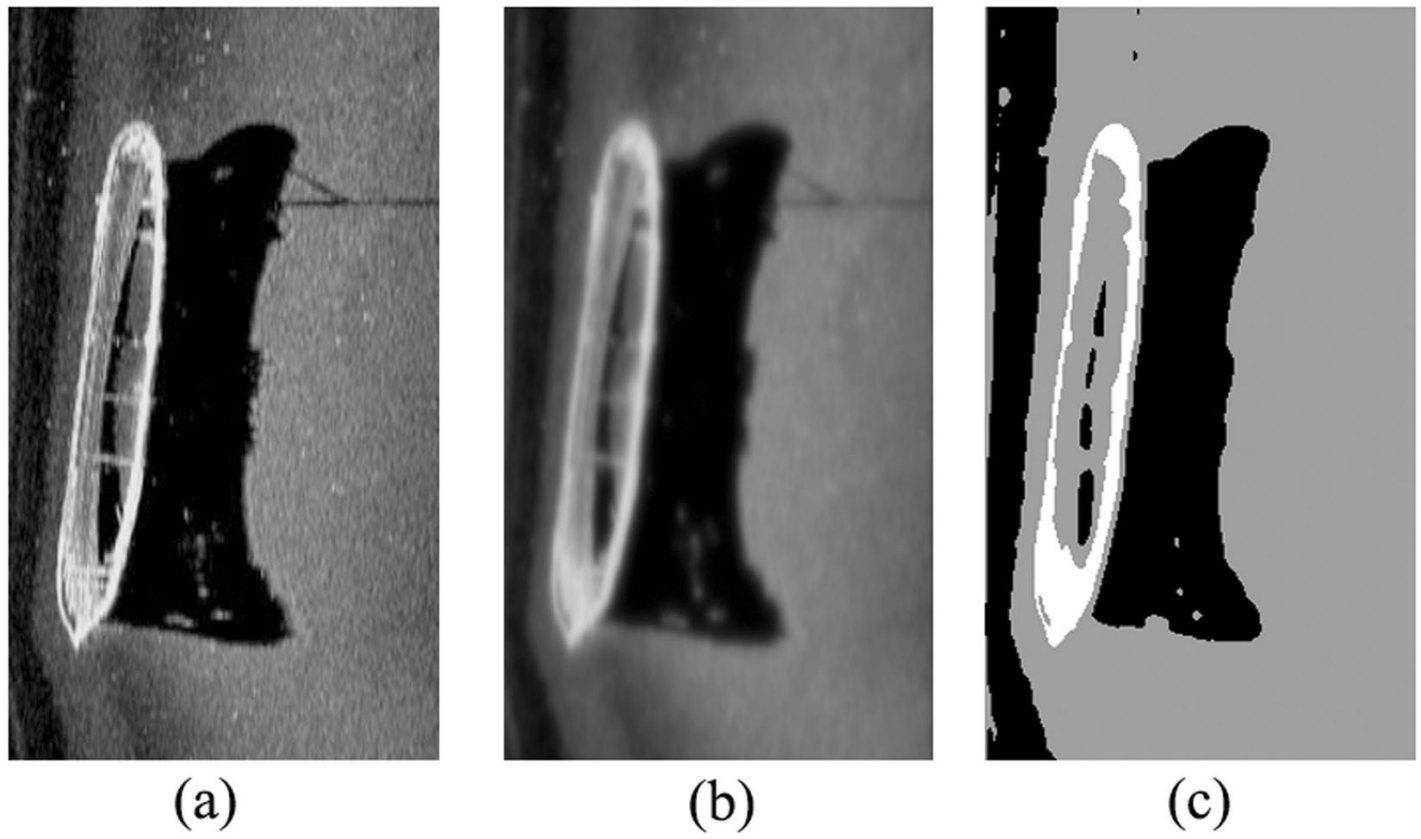
Detection results of original sonar image (image size: 393 × 218). (**a**) Original sonar image. (**b**) Smoothed image. (**c**) Detection result of ACA-IQPSO.

**Figure 12:**
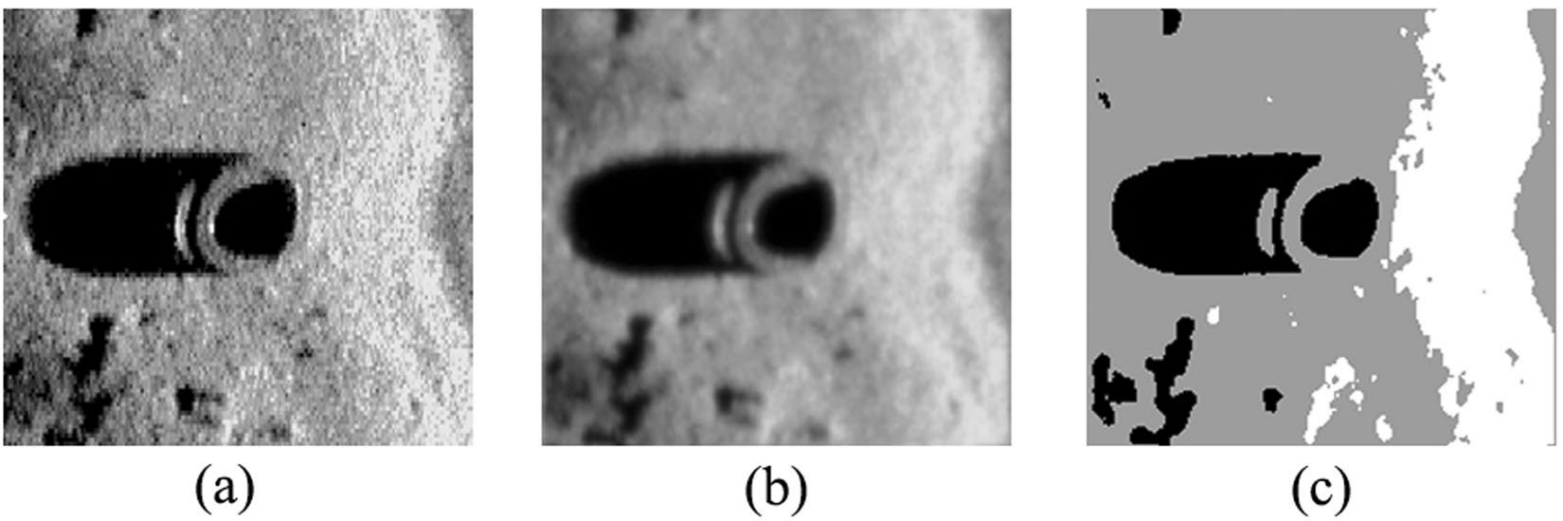
Detection results of original sonar image (image size: 197 × 211). (**a**) Original sonar image. (**b**) Smoothed image. (**c**) Detection result of ACA-IQPSO.

To further verify the adaptability of the proposed ACA-IQPSO in this paper, Fig. 10 shows the detection results of another original sonar image with floating objects. Fig. 11 shows the detection results of starboard original sonar image with ship. Fig. 12 shows the detection results of an original sonar image with bottom tire.

From the results shown in Figs 10, 11 and 12, the proposed ACA-IQPSO can better detect original sonar image with floating objects, partly buried objects and objects on the bottom in this paper, and it has a certain effectiveness and adaptability. Moreover, it provides better preconditions for the subsequent feature extraction and underwater object recognition.

### Experimental results and discussion of benchmark functions

To further verify the performance of the proposed ACA-IQPSO, 10 benchmark functions are used to compare the ACA-IQPSO with CA-IQPSO, CPSO, IQPSO, QPSO, and PSO. Among these benchmark functions, unimodal functions and multimodal functions are used to test local searching ability and global searching ability respectively. The details of benchmark functions are shown in Table 2.

**Table 2:** The details of benchmark functions.

Function	Name	Searching range	Global optimum	Dimension	Modality
F1	Sphere	[−5.12, 5.12]	0	10/30/50	unimodal
F2	Generalized Griewank	[−600, 600]	0	10/30/50	multimodal
F3	Stretched_V sine	[−10, 10]	0	10/30/50	multimodal
F4	Generalized Rastrigin’s	[−5.12, 5.12]	0	10/30/50	multimodal
F5	Generalized Rosenbrock’s	[−2.048, 2.048]	0	10/30/50	unimodal
F6	Quartic	[−1.28, 1.28]	0	10/30/50	unimodal
F7	Schwefel’s problem 2.22	[−10, 10]	0	10/30/50	unimodal
F8	Alpine	[−10, 10]	0	10/30/50	multimodal
F9	Salomon	[−100, 100]	0	10/30/50	multimodal
F10	Zakharov	[−5, 10]	0	10/30/50	unimodal

Minimum value, maximum value, mean value and standard deviation are obtained after each algorithm running 20 times. These values are used to evaluate the performance of algorithms in this paper. The relevant parameters of each run are as follows. The population size is 50, the maximum number of iterations is 50, and the dimensions are 10, 30 and 50 respectively. The acceleration coefficient is c1 = c2 = 2, the inertia weight is w = 0.8 in CPSO and PSO, size of cultural individuals is M = 15 in ACA-IQPSO, CA-IQPSO and CPSO, and the contraction-expansion coefficient linearly decreases from 1.0 to 0.5 in QPSO.

The performance of the algorithms is related to the dimension. When the dimension increases, the performance differences will be more significant. To analyse the performance of the algorithms in different dimensions, Table 3 shows the minimum value, maximum value, mean value and standard deviation of the different algorithms when the dimension is 10.

**Table 3:** Comparative results of different algorithm benchmark functions (10 dimension).

Fun	Eva	ACA-IQPSO	CA-IQPSO	CPSO	IQSO	QPSO	PSO
F1	min	0.001e-016	0.032	0.011	0.015	0.003	0.351
	max	3.391e-016	2.834	5.402	4.206	5.926	1.953
	mean	0.192e-016	0.958	1.203	1.042	1.458	1.079
	Std	0.754e-016	0.924	1.707	1.345	1.644	0.426
F2	min	0.001e-7	0.681	0.796	0.670	0.593	1.949
	max	2.986e-7	16.011	17.917	13.338	17.836	10.359
	mean	0.212e-7	4.046	5.221	3.379	4.634	5.001
	Std	0.709e-7	4.023	4.603	3.443	4.923	2.055
F3	min	3.933	9.215	6.677	6.543	8.427	8.899
	max	16.723	17.619	18.490	18.422	17.623	17.052
	mean	12.947	13.028	10.908	13.865	13.677	13.632
	Std	3.340	2.908	2.824	3.304	2.350	2.222
F4	min	0.004e-7	12.681	17.006	13.178	14.619	49.939
	max	5.933e-7	69.138	73.348	78.304	70.314	81.346
	mean	0.567e-7	38.767	36.329	44.271	46.230	66.521
	Std	1.434e-7	14.791	14.522	18.715	15.437	10.228
F5	min	2.649e-10	1.325e-009	6.888e-9	0.388	1.645	16.785
	max	4.215	6.986	6.680	74.023	62.363	101.409
	mean	0.533	2.578	1.755	18.506	22.168	35.422
	Std	1.184	2.164	2.238	25.789	20.582	24.832
F6	min	0.003e-37	2.394e-6	1.782e-5	2.569e-5	5.216e-5	1.253e-4
	max	8.433e-36	0.894	0.095	0.105	0.180	0.030
	mean	1.041e-36	0.078	0.023	0.015	0.042	0.007
	Std	2.004e-036	0.220	0.032	0.030	0.056	0.007
F7	min	0.241e-8	0.186	0.723	0.134	0.330	4.795
	max	3.673e-8	21.762	38.13	25.642	33.333	15.042
	mean	0.993e-8	7.362	9.168	9.518	12.018	9.494
	Std	0.795e-8	5.823	9.072	6.622	9.465	3.415
F8	min	0.011	0.785	0.984	9.407	10.707	9.575
	max	7.469	6.767	6.350	17.312	19.301	18.995
	mean	2.586	3.449	3.077	14.455	14.093	14.944
	Std	1.782	1.434	1.454	2.0588	2.704	2.467
F9	min	2.842e-16	15.831	5.857	0.427	6.068	0.168e + 3
	max	3.572e-11	2.743e + 3	2.346e + 3	2.290e + 3	2.125e + 3	0.911e + 3
	mean	2.385e-12	0.750 e + 3	0.570e + 3	0.474e + 3	0.571e + 3	0.451e + 3
	Std	7.875e-12	0.923 e + 3	0.697e + 3	0.665e + 3	0.631e + 3	0.183e + 3
F10	min	3.666e-13	14.815	7.553	1.650	13.058	20.870
	max	5.889e-6	120.323	94.352	101.203	75.740	340.018
	mean	3.091e-7	65.831	35.188	46.302	49.468	88.978
	Std	1.311e-6	25.901	22.231	27.809	15.914	74.781

Table 4 shows the minimum value, maximum value, mean value and standard deviation of the different algorithms when the dimension is 30.

**Table 4:** Comparative results of different algorithm benchmark functions (30 dimension).

Fun	Eva	ACA-IQPSO	CA-IQPSO	CPSO	IQSO	QPSO	PSO
F1	min	0.039e-15	0.426	3.022	0.807	1.483	12.816
	max	6.512e-15	43.273	92.352	38.831	42.565	63.996
	mean	1.359e-15	14.248	29.618	16.657	16.566	30.769
	Std	1.641e-15	10.955	25.664	10.757	12.917	12.267
F2	min	00.003e-10	2.779	19.372	4.260	13.011	52.608
	max	2.068e-10	96.362	218.610	166.551	124.224	188.514
	mean	0.150e-10	50.053	81.213	72.252	56.278	104.889
	Std	0.454e-10	29.201	218.620	47.163	31.852	40.233
F3	min	0.003	31.242	36.615	22.703	32.767	54.192
	max	0.008	70.987	71.181	70.508	71.353	76.834
	mean	0.005	55.235	54.264	53.203	53.933	68.053
	Std	0.002	10.980	8.427	12.734	10.160	6.381
F4	min	0.011e-11	113.299	109.928	163.012	171.195	251.666
	max	2.876e-11	265.058	287.250	262.475	312.808	342.299
	mean	0.491e-11	191.147	186.260	204.204	234.890	304.058
	Std	0.755e-11	46.833	39.916	34.786	38.261	23.294
F5	min	0.116	1.452	1.861	20.269	23.604	253.122
	max	20.526	17.469	14.236	294.183	386.263	1.938e + 3
	mean	9.896	7.640	7.447	118.545	146.226	818.542
	Std	6.325	3.799	3.155	55.172	79.135	462.124
F6	min	0.006e-32	0.004	1.782e-5	0.003	0.019	0.857
	max	3.368e-31	9.526	0.095	5.822	7.337	14.646
	mean	0.405e-31	1.853	0.023	1.163	1.391	4.836
	Std	0.788e-31	2.551	0.032	1.508	1.858	3.681
F7	min	0.484e-7	17.658	19.433	3.982	18.169	39.452
	max	3.909e-7	97.709	1.216e + 3	96.656	394.806	8.842e + 3
	mean	1.416e-7	52.346	110.072	47.747	67.819	1.343e + 3
	Std	0.847e-7	25.481	260.379	26.436	83.225	2.664e + 3
F8	min	4.566	4.629	3.721	8.549	6.546	7.514
	max	29.001	26.483	35.020	19.312	18.643	20.997
	mean	16.498	19.514	18.621	13.845	14.355	14.919
	Std	6.683	6.553	7.576	2.320	3.120	3.079
F9	min	1.160e-14	17.427	273.426	0.001	4.816	72.762
	max	5.880e-11	1823e + 4	2.092e + 4	634.916	750.721	1.332e + 3
	mean	7.973e-12	6.332e + 3	5.224e + 3	138.511	165.441	409.494
	Std	1.569e-11	5.626e + 3	5.019e + 3	185.771	187.897	257.137
F10	min	3.785e-7	201.443	59.370	4.303	4.763	10.925
	max	0.683	357.637	353.937	30.387	42.082	190.550
	mean	0.044	280.598	169.655	17.611	22.617	79.999
	Std	0.152	48.145	74.141	7.403	9.759	46.027

Table 5 shows the minimum value, maximum value, mean value and standard deviation of the different algorithms when the dimension is 50.

**Table 5:** Comparative results of different algorithm benchmark functions (50 dimension).

Fun	Eva	ACA-IQPSO	CA-IQPSO	CPSO	IQSO	QPSO	PSO
F1	min	0.079e-15	5.993	3.022	5.687	10.155	50.737
	max	6.226e-15	72.933	92.352	102.872	65.798	120.925
	mean	2.789e-15	33.574	29.618	32.045	31.107	83.588
	Std	1.763e-15	18.837	25.664	25.771	16.246	18.127
F2	min	0.003e-10	11.413	9.414	18.179	19.553	155.707
	max	1.255e-10	239.987	292.441	238.381	352.719	392.073
	mean	0.263e-10	143.819	102.649	96.500	130.760	276.319
	Std	0.344e-10	66.769	74.853	67.903	94.867	57.905
F3	min	0.007	60.837	76.891	38.798	56.907	108.870
	max	0.022	126.338	117.621	117.436	126.870	147.691
	mean	0.011	93.902	94.424	91.066	84.466	125.894
	Std	0.004	18.486	12.151	20.197	19.590	10.936
F4	min	0.011e-11	274.721	287.470	230.325	269.614	512.951
	max	1.199e-11	493.853	586.535	447.801	513.792	625.668
	mean	0.344e-11	381.700	361.106	359.192	388.933	558.881
	Std	0.332e-11	61.096	74.189	60.012	76.402	32.732
F5	min	1.081	5.610	2.703	21.911	0.062e + 3	812.024
	max	26.163	22.874	22.951	967.887	1.110e + 3	4.904e + 3
	mean	15.445	11.520	10.221	289.082	0.375e + 3	2.286e + 3
	Std	8.625	4.924	5.120	252.810	0.365e + 3	1.230e + 3
F6	min	0.001e-29	0.013	0.025	0.115	0.899	2.226
	max	0.540e-28	5.436	17.217	25.656	24.134	71.606
	mean	0.053e-28	1.916	3.834	4.258	5.964	30.829
	Std	0.121e-28	1.826	4.326	5.827	6.581	18.573
F7	min	0.161e-6	21.388	48.744	25.611	30.336	101.733
	max	1.009e-6	403.579	1.868e + 6	262.016	154.344	2.214e + 9
	mean	0.429e-6	114.307	8.917e + 4	86.307	80.571	2.212e + 8
	Std	0.211e-6	102.428	4.176e + 5	57.587	43.984	5.443e + 8
F8	min	12.171	20.174	15.978	102.736	90.465	8.113
	max	67.160	75.547	58.729	126.795	127.968	19.749
	mean	38.540	43.109	36.572	111.762	114.639	14.570
	Std	14.330	12.646	11.917	7.749	7.380	3.224
F9	min	1.167e-14	1.277e + 3	2.456e + 3	2.320	3.478	278.003
	max	1.252e-10	3.376e + 4	3.185e + 4	188.456	636.117	1.140e + 3
	mean	1.643e-11	1.253e + 4	1.316e + 4	47.756	113.181	530.507
	Std	3.053e-11	9.087e + 3	7.354e + 3	51.224	141.091	212.928
F10	min	1.548e-5	288.425	99.606	1.441	3.799	27.471
	max	26.913	927.776	465.631	22.398	33.833	157.421
	mean	1.492	552.816	297.274	11.694	17.970	83.039
	Std	5.977	168.856	110.946	6.713	7.287	42.022

From the comparative results in Table 3, Table 4 and Table 5, mean values of the IQPSO are smaller than the QPSO when the dimension is 10, 30 and 50, which indicates that the searching ability of the IQPSO is stronger than the QPSO. The standard deviation of the IQPSO and QPSO are close when dimension is 10, while standard deviation of the IQPSO are smaller than QPSO when dimension is 30 and 50. This demonstrates that the stability of the IQPSO is superior to QPSO in high dimension. Meanwhile, compared with the other algorithms, the proposed ACA-IQPSO is relatively closer to the global optimum value 0 after 50 iterations, which shows that the ACA-IQPSO has high convergence efficiency. In addition, minimum values, maximum values, mean values and standard deviation of the ACA-IQPSO in different benchmark functions are the smallest, which indicates that the proposed ACA-IQPSO has obvious advantages in searching ability and stability.

Similarly, Wilcoxon Signed-Rank Test and the Friedman test in nonparametric tests are adopted to further evaluate the performance of the algorithms by 10 benchmark functions in different dimensions^29,30^. Wilcoxon Signed-Rank Test is a paired comparison, which is used to compare the performance differences between two algorithms. If the p-values are less than or equal to the statistical significance value *τ*, the null hypothesis is rejected, which indicates that the two algorithms are different in performance. Friedman test is multiple comparison, which is used to compare the performance differences between more than two algorithms. When average rank is smaller, the performance of the algorithm is better.

Table 6 shows the p-values of Wilcoxon Signed-Rank Test by 10 benchmark functions in different dimensions (the statistical significance value *τ* = 0.05). Table 7 shows the average rank of Friedman Test by 10 benchmark functions in different dimensions.

**Table 6:** The p-values of Wilcoxon Signed-Rank Test.

Dimension	ACA-IQPSO vs CA-IQPSO	ACA-IQPSO vs CPSO	ACA-IQPSO vs IQPSO	ACA-IQPSO vs QPSO
10	0.0020	0.0195	0.0020	0.0020
30	0.0059	0.0098	0.0059	0.0059
50	0.0059	0.0137	0.0020	0.0020

**Table 7:** The average rank of Friedman Test.

Dimension	ACA-IQPSO	CA-IQPSO	CPSO	IQPSO	QPSO	PSO
10	1.1	3.6	3.1	3.8	4.9	4.5
30	1.5	3.9	4.0	2.8	3.5	5.3
50	1.0	4.3	4.0	2.8	3.5	5.4

As seen in Table 6 and Table 7, the p-values between the ACA-IQPSO and other algorithms are smaller than the statistical significance value, which shows the significant performance differences between the ACA-IQPSO and other algorithms. Meanwhile, the average ranks of the ACA-IQPSO are smaller than the other algorithms. It can indicate that the performance of the ACA-IQPSO is superior to other algorithms. Therefore, through the analysis of benchmark functions, it can demonstrate that the proposed ACA-IQPSO is obviously better than other algorithms in searching ability, convergence efficiency and stability.

## Conclusions

Considering the growing requirements of underwater sonar image detection, this paper proposed the ACA-IQPSO to detect underwater sonar images In the population space, iterative times and the fitness value of particles are used as factors to adaptively adjust the contraction-expansion coefficient of the QPSO. IQPSO can make particles adjust their behaviour to improve searching ability. In belief space, a new update strategy is adopted to update the cultural individuals according to the update strategy in SFLA. Moreover, to enhance utilization of information in population and belief spaces, accept function and influence function are redesigned in the new communication protocol. The new communication protocol can make belief space with adequate evolutionary information that can more precisely guide the evolution of particles in the population space and further improve the searching ability of the algorithm. Furthermore, the new communication protocol can enhance convergence efficiency of algorithm.

The proposed ACA-IQPSO is based on a clustering model. The object-highlight, shadow and background regions in the sonar image are detected by clustering centres. The experimental results demonstrate that ACA-IQPSO can locate good clustering centers and accurately complete underwater objects detection. Compared with other algorithms, the proposed ACA-IQPSO has good effectiveness and adaptability, and it has powerful searching ability and high convergence efficiency. Meanwhile, the performance of the proposed ACA-IQPSO is further demonstrated by the analysis of benchmark functions, it can show that the proposed ACA-IQPSO is significantly better than the other algorithms in searching ability, convergence efficiency and stability. Therefore, the proposed method can provide better preconditions for the subsequent feature extraction and underwater object recognition. It has important theoretical and practical value.
